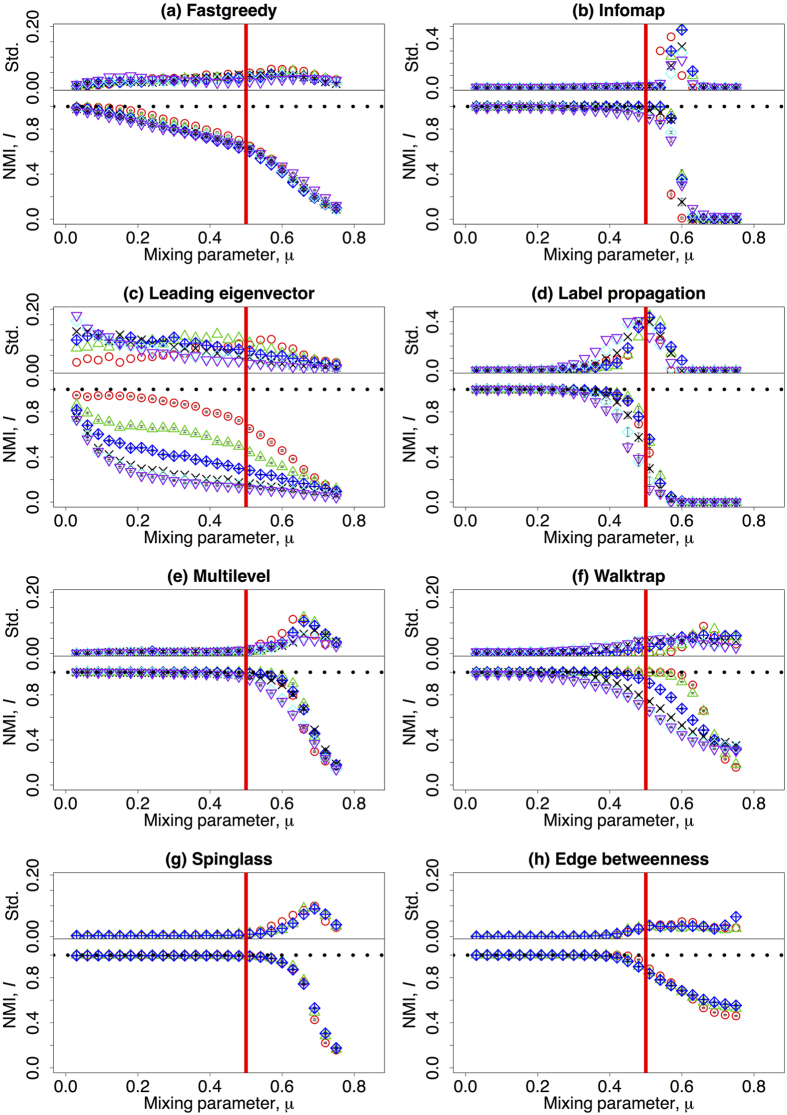# Corrigendum: A Comparative Analysis of Community Detection Algorithms on Artificial Networks

**DOI:** 10.1038/srep46845

**Published:** 2017-06-26

**Authors:** Zhao Yang, René Algesheimer, Claudio J. Tessone

Scientific Reports 6: Article number: 30750; 10.1038/srep30750 published online: 08 01 2016; updated: 06 26 2017.

This Article contained errors.

Figure 1f incorrectly showed curves for the values N = 233, 279, 335, 402, 482, and 579.

The correct Figure 1 appears below.

The Results section contained a typographical error under the subheading “The role of network size”.

“Most of the algorithms can well uncover the communities when *μ* ⪆ 0.2”.

now reads:

“Most of the algorithms can well uncover the communities when *μ* ⪅ 0.2”.

The Supplementary Information file originally published with this Article contained errors in the f panels of Supplementary Figures 1, 2, 3, 4, and 5, where these panels incorrectly showed curves for the values N = 233, 279, 335, 402, 482, and 579.

These errors have now been corrected in the PDF and HTML versions of this Article.

## Figures and Tables

**Figure 1:**