# Supplementary material for: A Comparative Analysis of Community Detection Algorithms on Artificial Networks
Source: Sci Rep. 2016 Aug 1;6:30750. doi: 10.1038/srep30750 (PMC4967864; doi:10.1038/srep30750)
Supplement: Supplementary Information [file srep30750-s1.pdf]

Supplementary information: A Comparative Analysis of Community Detection Algorithms on Artificial Networks

Zhao Yang[1,*], René Algesheimer[1], and Claudio J. Tessone[1]

[1]URPP Social Networks, University of Zürich, Andreasstrasse 15, CH-8050 Zürich, Switzerland
[*]zhao.yang@business.uzh.ch

ABSTRACT

In this “Supplementary Information”, we report extended results for different normalisation procedures of the mutual information. We show that the results display a similar behavior regardless of the specific normalisation way chosen.

Different normalization methods for mutual information

The accuracy of different community detection algorithms can be evaluated by the *normalised mutual information*[1]. As it has been pointed out by Vinh *et al.*, there exist five different normalised versions of the mutual information[2]: I_{joint} $(=\frac{i(\mathscr{P},\bar{\mathscr{P}})}{H(\mathscr{P},\bar{\mathscr{P}})})$, I_{max} $(=\frac{i(\mathscr{P},\bar{\mathscr{P}})}{max\{H(\mathscr{P}),H(\bar{\mathscr{P}})\}})$, I_{sum} $(=\frac{i(\mathscr{P},\bar{\mathscr{P}})}{\frac{1}{2}(H(\mathscr{P})+H(\bar{\mathscr{P}}))})$, I_{sqrt} $(=\frac{i(\mathscr{P},\bar{\mathscr{P}})}{\sqrt{H(\mathscr{P})H(\bar{\mathscr{P}})}})$, and I_{min} $(=\frac{i(\mathscr{P},\bar{\mathscr{P}})}{min\{H(\mathscr{P}),H(\bar{\mathscr{P}})\}})$. Different normalisation methods are sensitive to different partition properties and have different theoretical properties.

In this “Supplementary information”, we show the effect of the mixing parameter and network size on all five different NMIs and conclude that the results are similar to each other. In the main text, we report the results of I_{sum}[2], which is consistent with Danon *et al.*[1].

0.1 The role of the network mixing parameter on accuracy

In Figure 1, 2, 3, 4, and 5, we show the effect of the mixing parameter on I_{joint}, I_{max}, I_{sum}, I_{sqrt}, and I_{min}, separately. The detailed explanation of the plot I_{sum} can be found in the main text. Comparing different figures, we conclude that: (1) I_{joint} provides the smallest values and I_{min} provides the largest ones, and (2) all the NMIs display similar patterns.

0.2 The role of network size on accuracy

In Figure 6, 7, 8, 9, and 10, we show the effect of the network size on I_{joint}, I_{max}, I_{sum}, I_{sqrt}, and I_{min}, separately. Comparing the different plots we get the same conclusion as before.

References

1. Danon, L., Diaz-Guilera, A., Duch, J. & Arenas, A. Comparing community structure identification. *Journal of Statistical Mechanics: Theory and Experiment* **2005**, P09008 (2005).

2. Vinh, N. X., Epps, J. & Bailey, J. Information theoretic measures for clusterings comparison: Variants, properties, normalization and correction for chance. *The Journal of Machine Learning Research* **11**, 2837–2854 (2010).

Figure 1. (lower row) The mean value of I_{joint} dependent on the mixing parameter μ. (upper row) The standard deviation of I_{joint} dependent on μ.

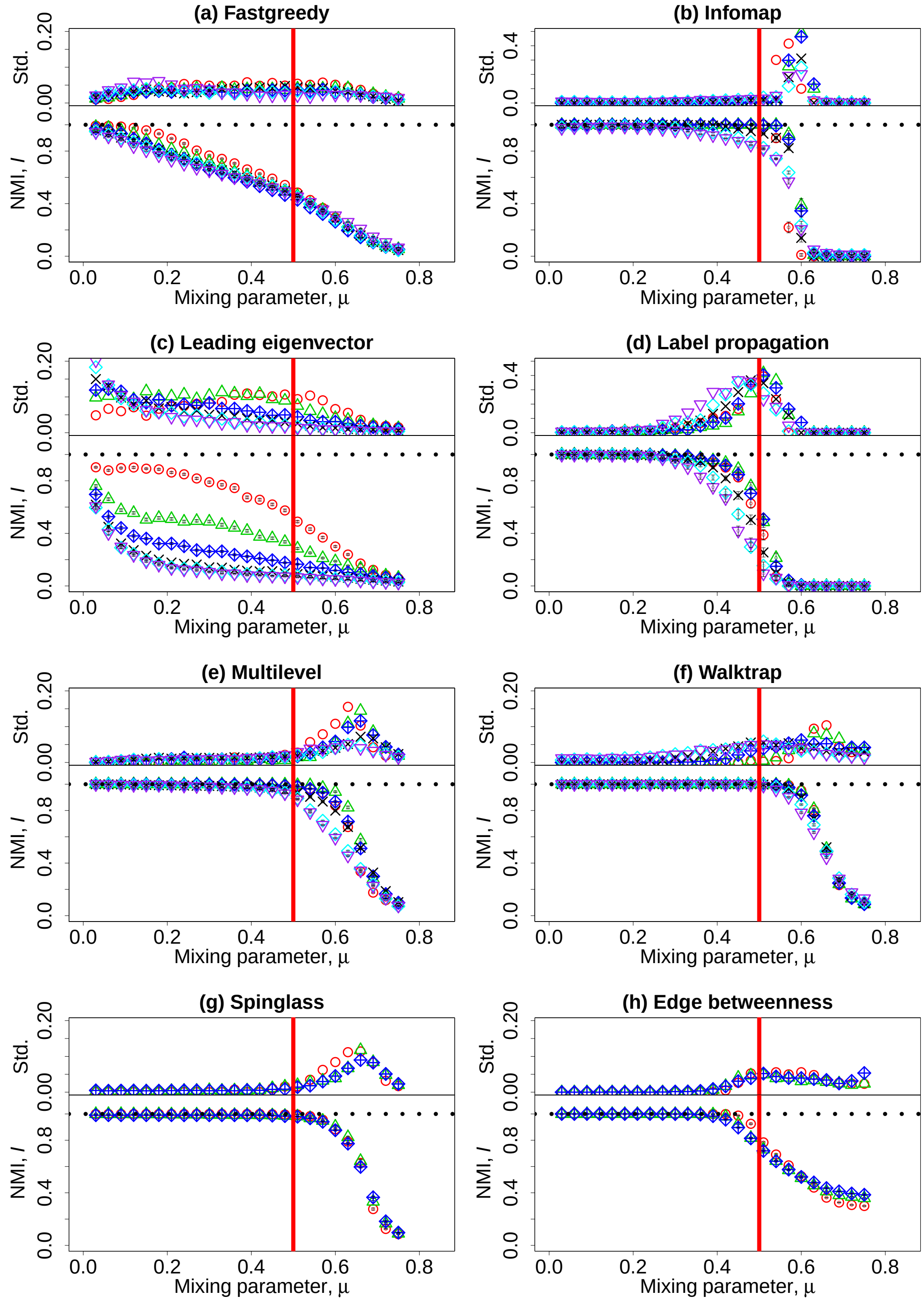

Different colours refer to different number of nodes: red ($N = 233$), green ($N = 482$), blue ($N = 1000$), black ($N = 3583$), cyan ($N = 8916$), and purple ($N = 22186$). Please notice that the vertical axis on the subfigures might have different scale ranges. The vertical red line corresponds to the strong definition of community where $\mu = 0.5$. The horizontal black dotted line corresponds to $I = 1$. The other parameters are described in the main text.

Figure 2. (lower row) The mean value of I_{max} dependent on the mixing parameter μ. (upper row) The standard deviation of I_{max} dependent on μ.

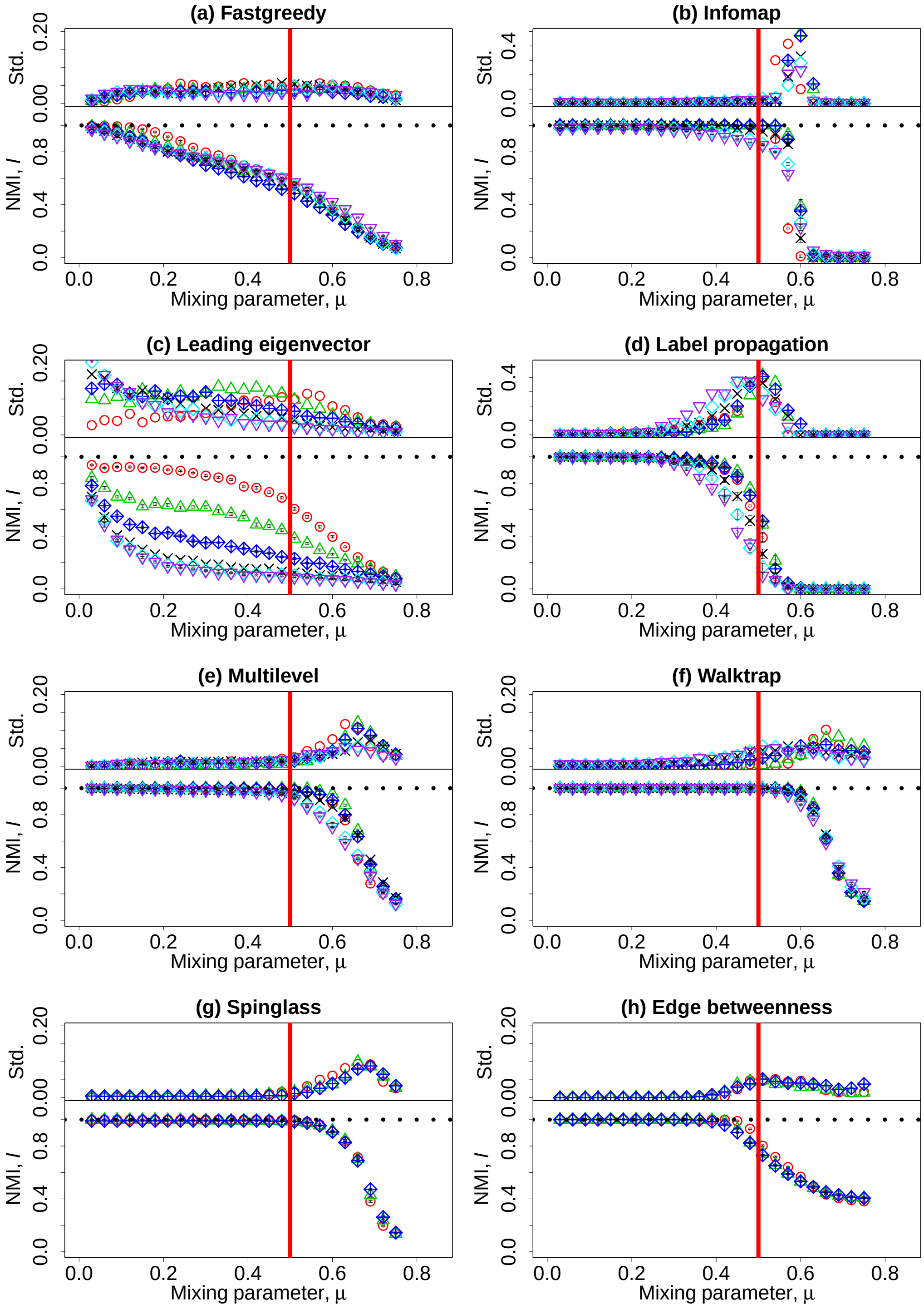

Different colours refer to different number of nodes: red ($N = 233$), green ($N = 482$), blue ($N = 1000$), black ($N = 3583$), cyan ($N = 8916$), and purple ($N = 22186$). Please notice that the vertical axis on the subfigures might have different scale ranges. The vertical red line corresponds to the strong definition of community where $\mu = 0.5$. The horizontal black dotted line corresponds to $I = 1$. The other parameters are described in the main text.

Figure 3. (lower row) The mean value of I_{sum} dependent on the mixing parameter μ. (upper row) The standard deviation of I_{sum} dependent on μ.

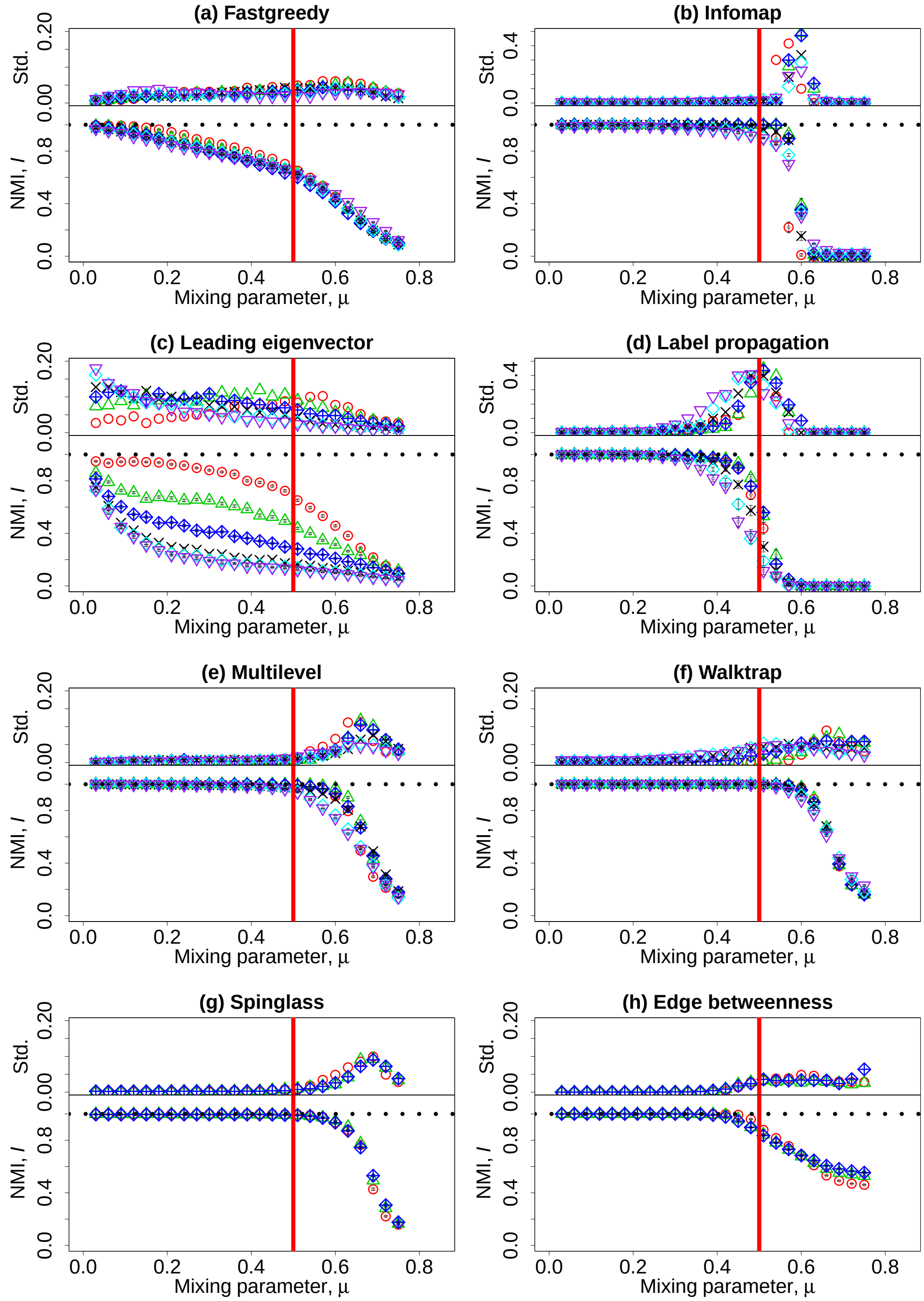

Different colours refer to different number of nodes: red ($N = 233$), green ($N = 482$), blue ($N = 1000$), black ($N = 3583$), cyan ($N = 8916$), and purple ($N = 22186$). Please notice that the vertical axis on the subfigures might have different scale ranges. The vertical red line corresponds to the strong definition of community where $\mu = 0.5$. The horizontal black dotted line corresponds to $I = 1$. The other parameters are described in the main text.

Figure 4. (lower row) The mean value of I_{sqrt} dependent on the mixing parameter μ. (upper row) The standard deviation of I_{sqrt} dependent on μ.

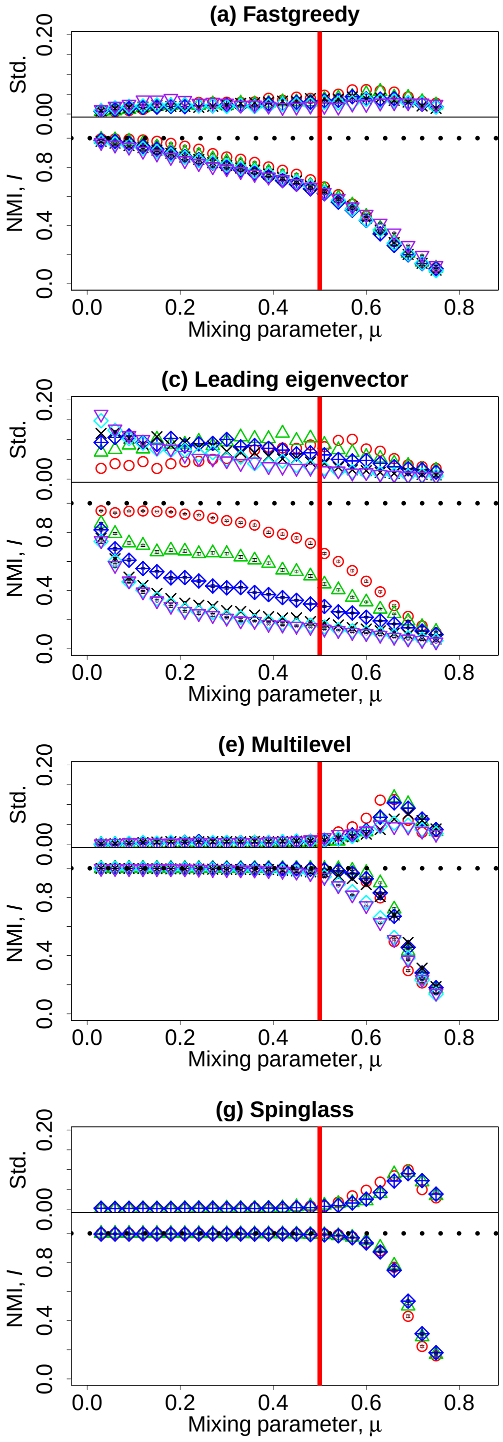

Different colours refer to different number of nodes: red ($N = 233$), green ($N = 482$), blue ($N = 1000$), black ($N = 3583$), cyan ($N = 8916$), and purple ($N = 22186$). Please notice that the vertical axis on the subfigures might have different scale ranges. The vertical red line corresponds to the strong definition of community where $\mu = 0.5$. The horizontal black dotted line corresponds to $I = 1$. The other parameters are described in the main text.

Figure 5. (lower row) The mean value of I_{min} dependent on the mixing parameter μ. (upper row) The standard deviation of I_{min} dependent on μ.

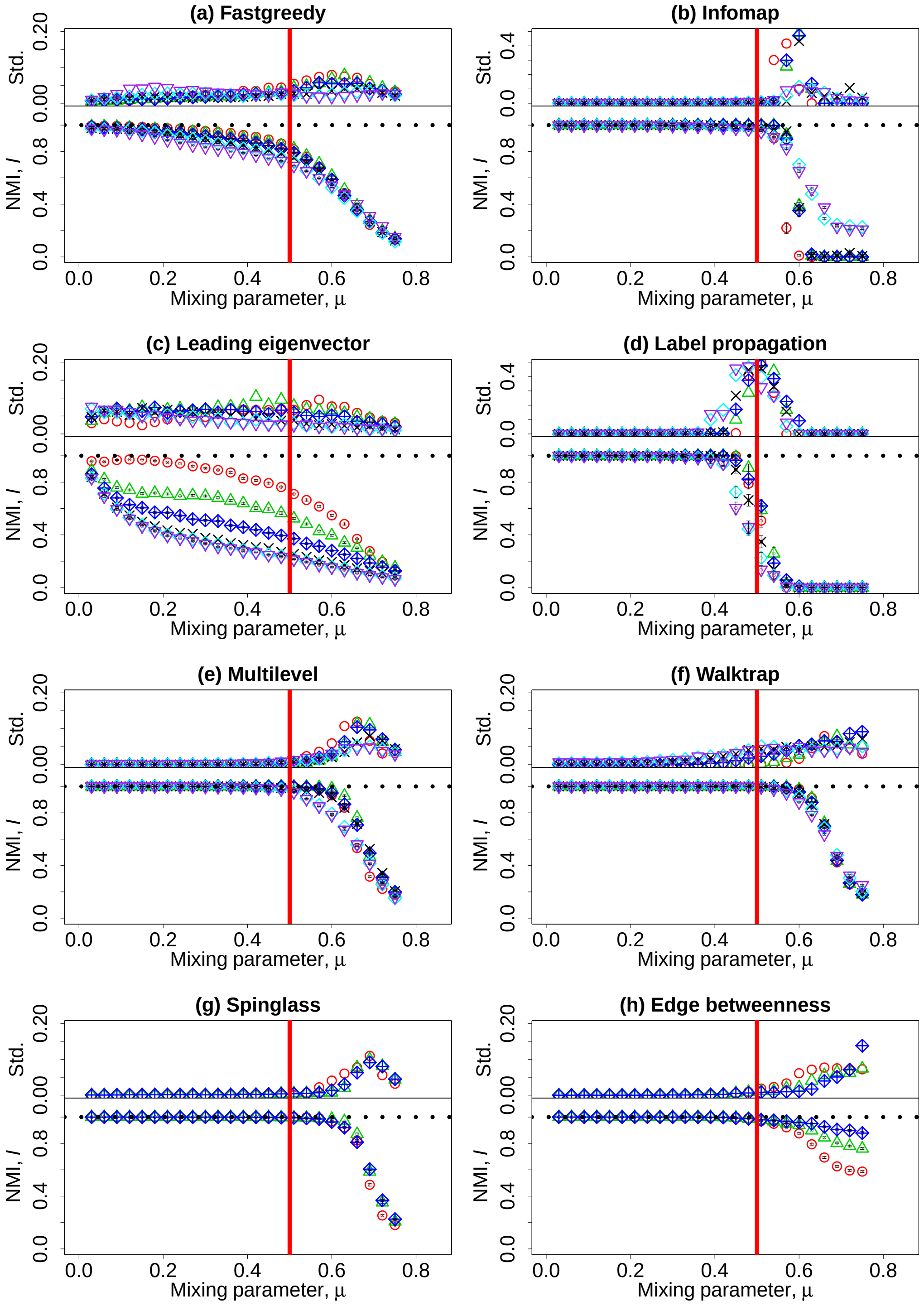

Different colours refer to different number of nodes: red ($N = 233$), green ($N = 482$), blue ($N = 1000$), black ($N = 3583$), cyan ($N = 8916$), and purple ($N = 22186$). Please notice that the vertical axis on the subfigures might have different scale ranges. The vertical red line corresponds to the strong definition of community where $\mu = 0.5$. The horizontal black dotted line corresponds to $I = 1$. The other parameters are described in the main text.

Figure 6. (lower row) The mean value of I_{joint} dependent on the number of nodes N in the benchmark graphs on a *linear-log* scale. (upper row) The standard deviation of I_{joint} dependent on N on a *linear-log* scale.

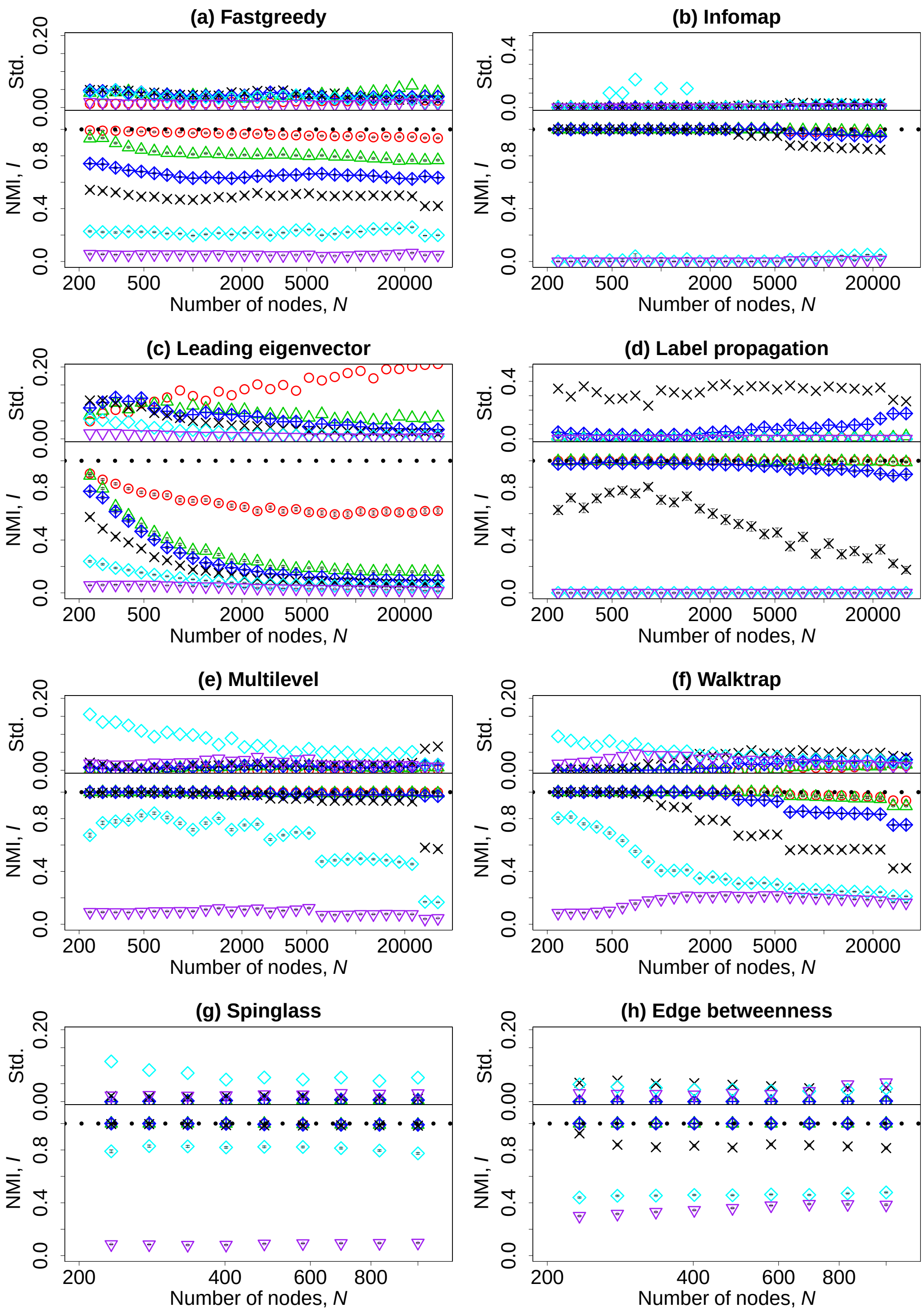

Different colours refer to different values of the mixing parameter: red ($\mu = 0.03$), green ($\mu = 0.18$), blue ($\mu = 0.33$), black ($\mu = 0.48$), cyan ($\mu = 0.63$), and purple ($\mu = 0.75$). Please notice that the vertical axis on the subfigures might have different scale ranges. The horizontal black dotted line corresponds to $I = 1$. Due to the computing speed, Spinglass and Edge betweenness algorithms have been tested only on networks with $N \leq 1000$, and Infomap algorithm has been tested on networks with $N \leq 22186$. The other parameters are described in the main text.

Figure 7. (lower row) The mean value of I_{max} dependent on the number of nodes N in the benchmark graphs on a *linear-log* scale. (upper row) The standard deviation of I_{max} dependent on N on a *linear-log* scale.

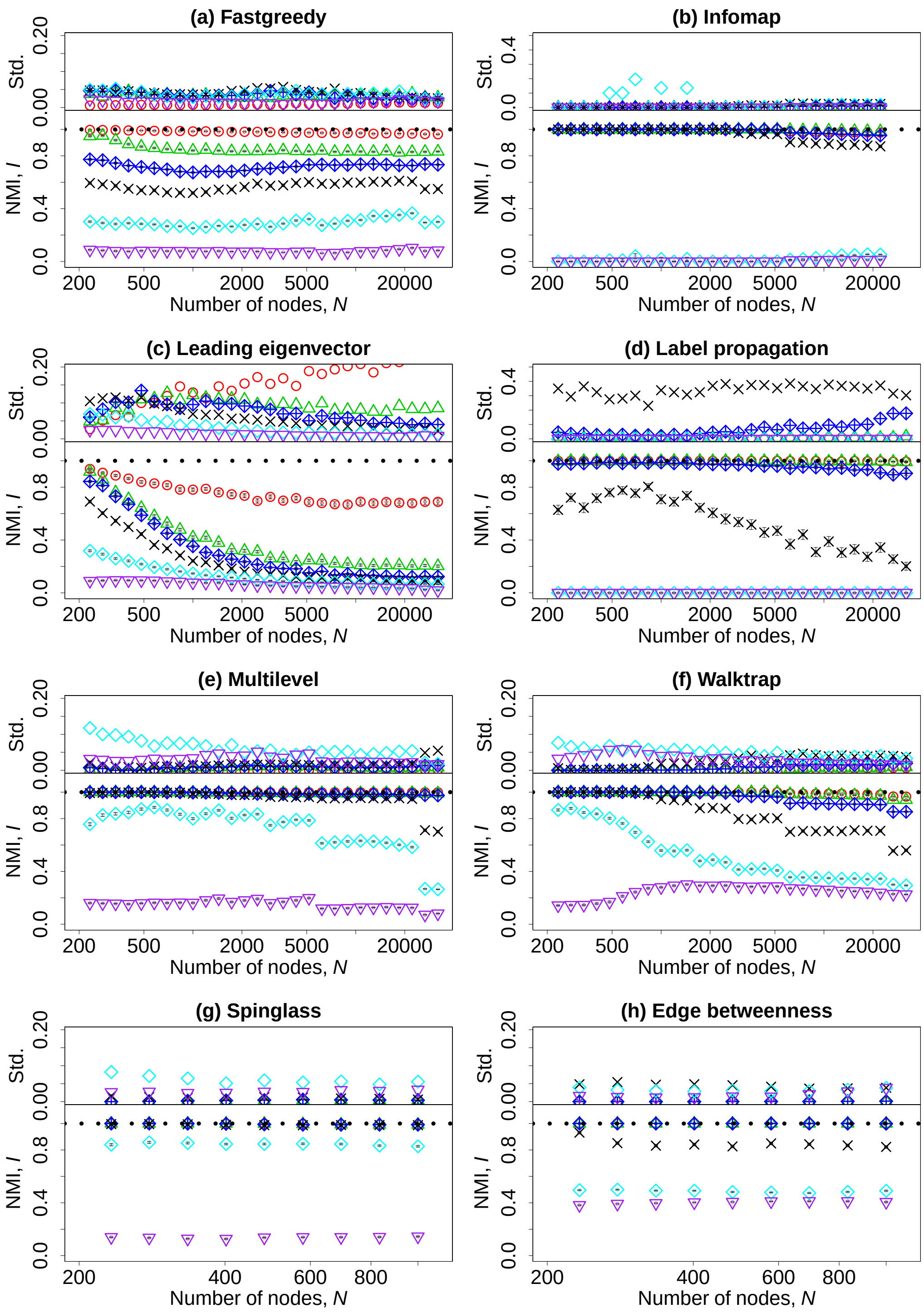

Different colours refer to different values of the mixing parameter: red ($\mu = 0.03$), green ($\mu = 0.18$), blue ($\mu = 0.33$), black ($\mu = 0.48$), cyan ($\mu = 0.63$), and purple ($\mu = 0.75$). Please notice that the vertical axis on the subfigures might have different scale ranges. The horizontal black dotted line corresponds to $I = 1$. Due to the computing speed, Spinglass and Edge betweenness algorithms have been tested only on networks with $N \leq 1000$, and Infomap algorithm has been tested on networks with $N \leq 22186$. The other parameters are described in the main text.

Figure 8. (lower row) The mean value of I_{sum} dependent on the number of nodes N in the benchmark graphs on a *linear-log* scale. (upper row) The standard deviation of I_{sum} dependent on N on a *linear-log* scale.

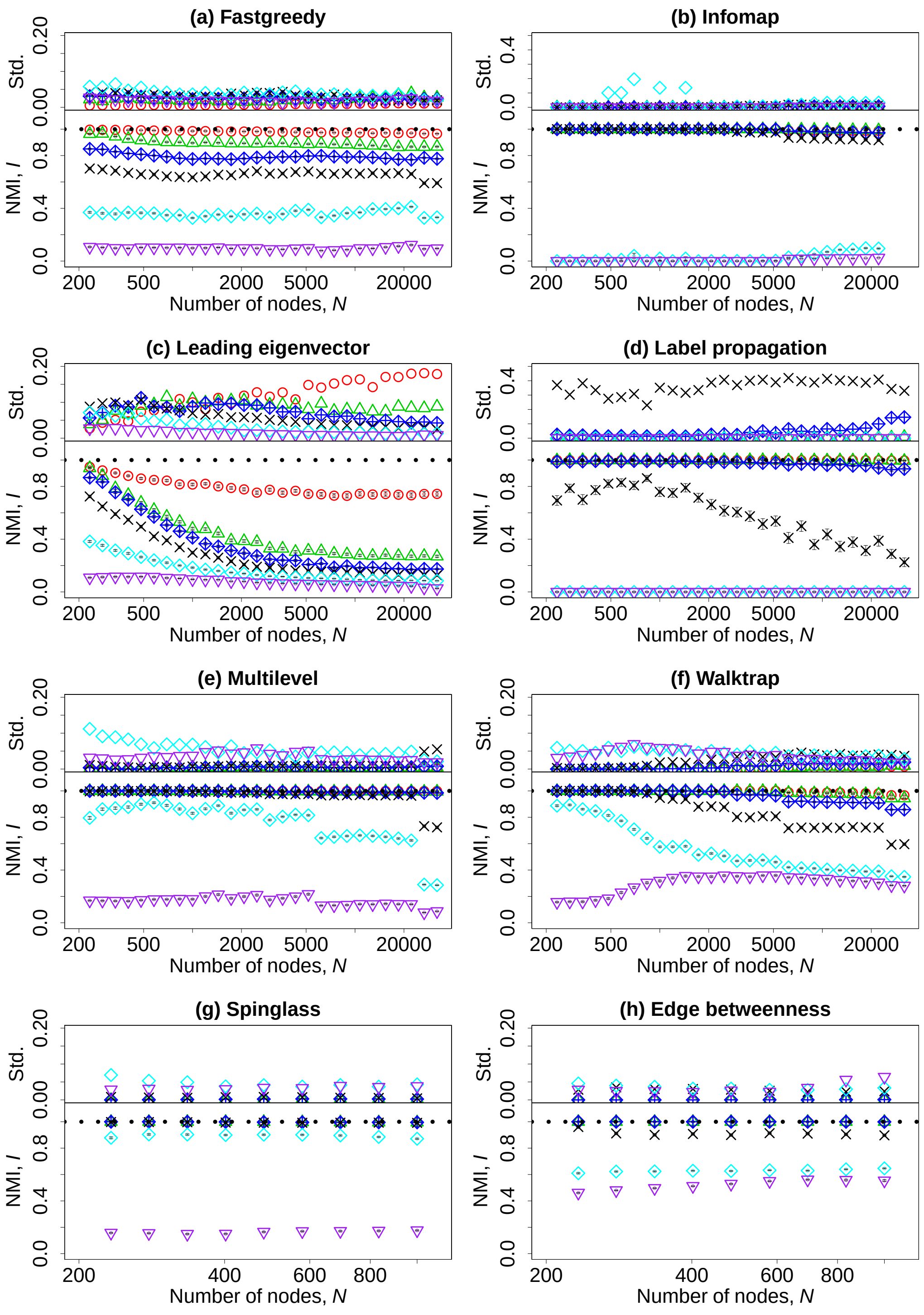

Different colours refer to different values of the mixing parameter: red ($\mu = 0.03$), green ($\mu = 0.18$), blue ($\mu = 0.33$), black ($\mu = 0.48$), cyan ($\mu = 0.63$), and purple ($\mu = 0.75$). Please notice that the vertical axis on the subfigures might have different scale ranges. The horizontal black dotted line corresponds to $I = 1$. Due to the computing speed, Spinglass and Edge betweenness algorithms have been tested only on networks with $N \leq 1000$, and Infomap algorithm has been tested on networks with $N \leq 22186$. The other parameters are described in the main text.

Figure 9. (lower row) The mean value of I_{sqrt} dependent on the number of nodes N in the benchmark graphs on a *linear-log* scale. (upper row) The standard deviation of I_{sqrt} dependent on N on a *linear-log* scale.

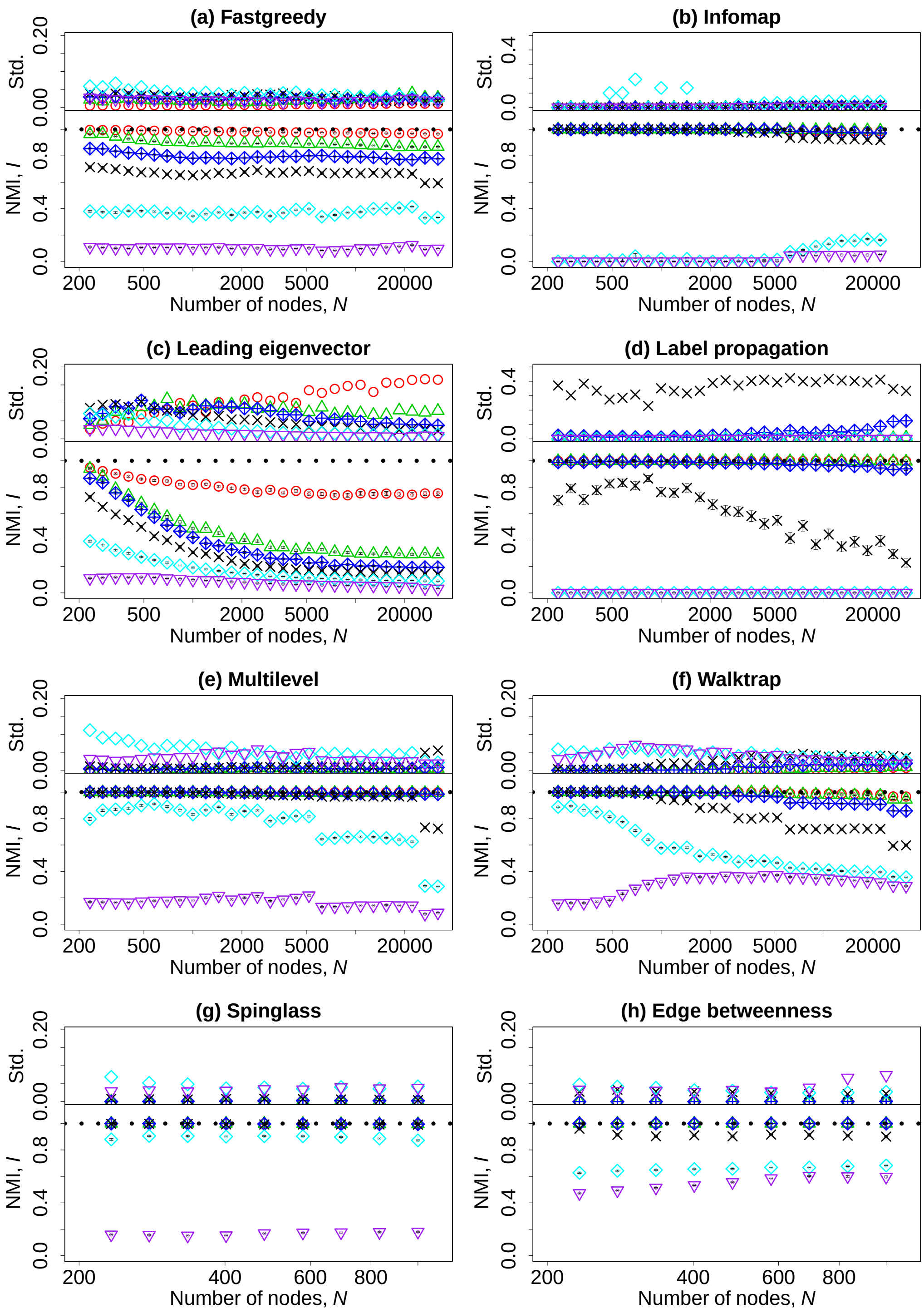

Different colours refer to different values of the mixing parameter: red ($\mu = 0.03$), green ($\mu = 0.18$), blue ($\mu = 0.33$), black ($\mu = 0.48$), cyan ($\mu = 0.63$), and purple ($\mu = 0.75$). Please notice that the vertical axis on the subfigures might have different scale ranges. The horizontal black dotted line corresponds to $I = 1$. Due to the computing speed, Spinglass and Edge betweenness algorithms have been tested only on networks with $N \leq 1000$, and Infomap algorithm has been tested on networks with $N \leq 22186$. The other parameters are described in the main text.

Figure 10. (lower row) The mean value of I_{min} dependent on the number of nodes N in the benchmark graphs on a *linear-log* scale. (upper row) The standard deviation of I_{min} dependent on N on a *linear-log* scale.

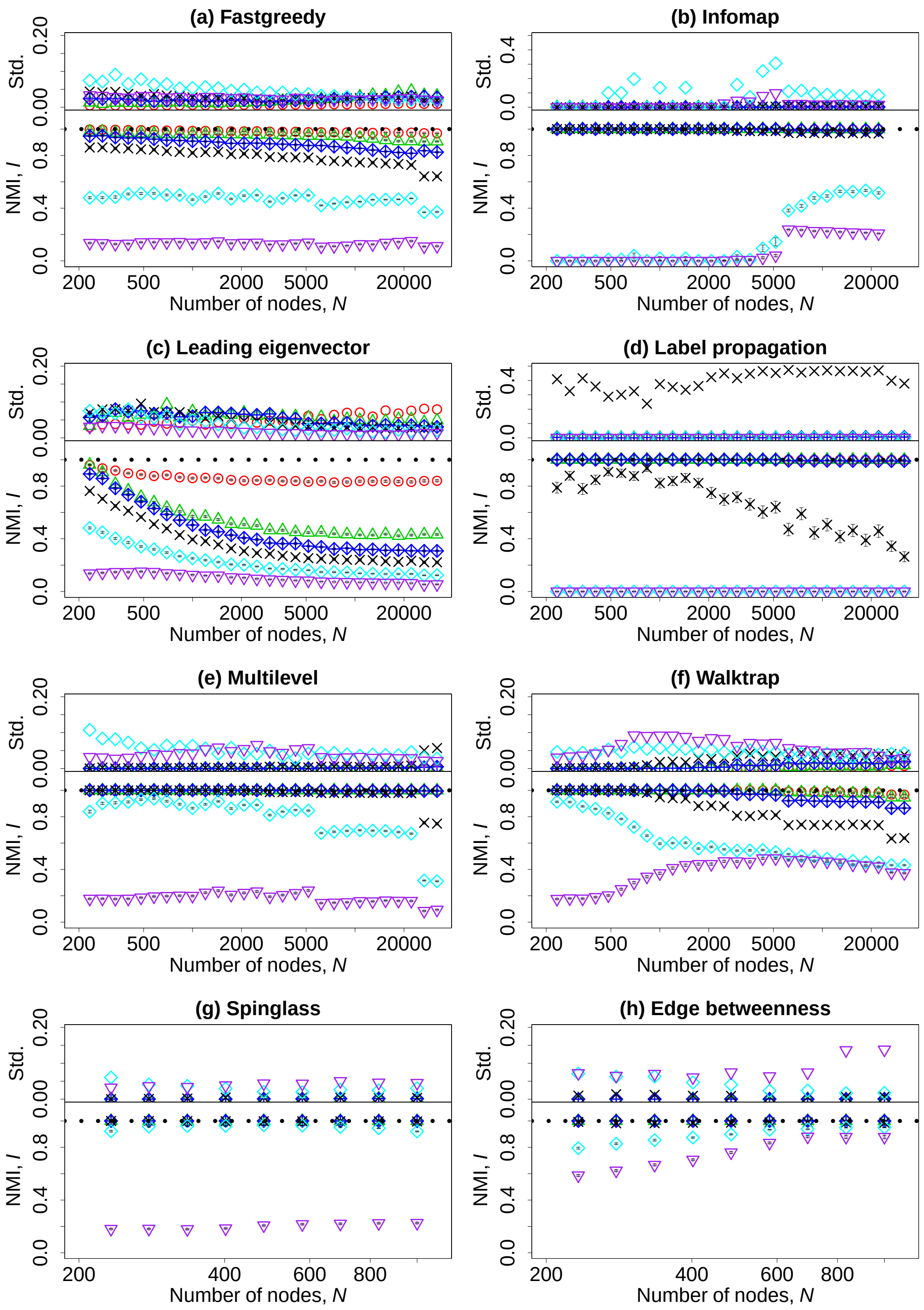

Different colours refer to different values of the mixing parameter: red ($\mu = 0.03$), green ($\mu = 0.18$), blue ($\mu = 0.33$), black ($\mu = 0.48$), cyan ($\mu = 0.63$), and purple ($\mu = 0.75$). Please notice that the vertical axis on the subfigures might have different scale ranges. The horizontal black dotted line corresponds to $I = 1$. Due to the computing speed, Spinglass and Edge betweenness algorithms have been tested only on networks with $N \leq 1000$, and Infomap algorithm has been tested on networks with $N \leq 22186$. The other parameters are described in the main text.
